# In Vivo Evaluation of Decellularized Human Tooth Scaffold for Dental Tissue Regeneration

**DOI:** 10.3390/app11188472

**Published:** 2021-09-13

**Authors:** Ik-Hwan Kim, Mijeong Jeon, Kyounga Cheon, Sun Ha Kim, Han-Sung Jung, Yooseok Shin, Chung Min Kang, Seong-Oh Kim, Hyung-Jun Choi, Hyo-Seol Lee, Ko Eun Lee, Je Seon Song

**Affiliations:** 1 Department of Pediatric Dentistry, College of Dentistry, Yonsei University, Seoul 03722, Korea; 2 Oral Science Research Center, College of Dentistry, Yonsei University, Seoul 03722, Korea; 3 Department of Pediatric Dentistry, University of Alabama at Birmingham, Birmingham, AL 35294, USA; 4 Division in Anatomy & Developmental Biology, Department of Oral Biology, College of Dentistry, Yonsei University, Seoul 03722, Korea; 5 Department of Conservative Dentistry, College of Dentistry, Yonsei University, Seoul 03722, Korea; 6 Department of Pediatric Dentistry, Kyung Hee University Dental Hospital, Seoul 02447, Korea; 7 Institute of Craniofacial Deformity, College of Dentistry, Yonsei University, Seoul 03722, Korea

**Keywords:** decellularized tooth scaffold, dental pulp stem cell, periodontal ligament stem cell, tissue regeneration

## Abstract

Conventional root canal treatment may result in loss of tooth vitality, which can lead to unfavorable treatment outcomes. Notably, a ceased tooth development of immature permanent teeth with open apices, regeneration of periodontal ligaments (PDL), and pulp is highly expected healing process. For regeneration, the scaffold is one of the critical components that carry biological benefits. Therefore, this study evaluated a decellularized human tooth as a scaffold for the PDL and pulp tissue regeneration. A tooth scaffold was fabricated using an effective decellularization method as reported in previous studies. PDL stem cells (PDLSCs) and dental pulp stem cells (DPSCs) obtained from human permanent teeth were inoculated onto decellularized scaffolds, then cultured to transplant into immunosuppressed mouse. After 9 weeks, PDLSCs and DPSCs that were inoculated onto decellularized tooth scaffolds and cultured in an in vivo demonstrated successful differentiation. In PDLSCs, a regeneration of the cementum/PDL complex could be expected. In DPSCs, the expression of genes related to revascularization and the hard tissue regeneration showed the possibility of pulp regeneration. This study suggested that the potential possible application of decellularized human tooth could be a scaffold in regeneration PDL and pulp tissue along with PDLSCs and DPSCs, respectively, as a novel treatment method.

## 1. Introduction

The tooth is composed of enamel, dentin, cementum, pulp, and periodontal ligaments (PDL). Although tooth is confined with hard and calcified structures (enamel and dentin), dental pulp, and PDL are vulnerable to damages caused by dental caries, infections, and trauma [1]. The aim of the conventional treatment for a tooth is removal of affected/infected tissue and bacteria, and replacement with inert or synthetic dental materials. However, this approach may result in loss of biological function and cessation of root development of immature permanent teeth, which will eventually cause the tooth to become susceptible to secondary infections, post-operative fractures, and loss of biological function [2,3]. One of the promising alternatives to the conventional treatment is concept of PDL and pulp regeneration [4–8]. Current concept of regeneration is composed of important factors such as cells, scaffolds, and growth factors [9,10]. Stem cells (SCs) have the potential to proliferate, self-renew, and differentiate into a variety of functional cells [11]. Dental SCs (DSCs) are post-natal stem cells that have mesenchymal stem cell-like qualities, including the capacity for self-renewal and multi-lineage differentiation [12]. DSCs can be collected from craniofacial bones, dental follicles, tooth germs, dental pulp, PDL, apical papillae, oral mucosa, gingiva, and the periosteum [13]. Various DSCs are being studied for their therapeutic potential [12] including tooth repair [14].

Stem cells can play a role in tissue regeneration, but the cell conditions in the laboratory environment and in the original tissue are different. Various types of scaffolds are used in regenerative tissue engineering to enhance stem cell differentiation, proliferation, and metabolism [15]. Scaffolds could promote stem cell adhesion and proliferation by mimicking the microenvironments and supporting structures of tissues [16,17]. Although more tissue regeneration research is being conducted nowadays, tissue repairs are still more common than tissue regeneration. After injection of suspended stem cells, it is almost impossible to control cell location and function, so a suitable scaffold is required. In particular, extracellular matrix (ECM) is important for scaffold fabrication and selection for reasons such as biological compatibility, biological degradability, and the possibility of rapid remodeling in vivo [18]. Synthetic scaffold using CAD/CAM technologies, laser-assisted bioprinting technologies, and a scaffold using decellularized organs are being studied to improve the effectiveness of tissue regeneration [19,20] including dental tissue regeneration [21]. Among the available dental scaffolds, decellularized tissues, decellularized human tooth, and tooth bud scaffolds were studied as a promising dental tissue regeneration scaffolds in current studies [22–24]. Moreover, the results of dental tissue regeneration are affected by the delivery of growth factors. Studies on regeneration associated with the interaction between the dental or nondental stem cells and the growth factors under in vivo environment have been recently reported [2,25,26]. Various growth factors regulate transplanted or endogenously homed cells, and play a critical role in cell proliferation, odontoblastic differentiation, extracellular matrix synthesis, dentinogenesis, and chemotaxis [27–31].

The periodontium is a supportive complex of gingiva, PDL, cementum, and alveolar bone, which provides attachment to the bone and acts as a barrier from the oral micropathogen. Secondarily, PDLs are directly associated with tooth vitality [32]. However, dental infection and trauma may bring significant adverse effects to the pulp and PDL, ultimately causing poor regeneration capability of the PDL. Several studies attempted periodontal ligament regeneration by utilizing PDLSCs and scaffolds including cell sheets [33,34].

Pulp regeneration is important in restoring tooth vitality and maintaining the development of immature permanent teeth. Innovative regenerative endodontic procedures (REP) can overcome the limitations of conventional root canal treatments [35,36]. However, REPs form dental pulp-like tissue, but in situ transplantation of human dental pulp stem cells (DPSC) has been shown to successfully regenerate vascularized pulp tissue [37,38]. The direct transplantation of stem cells produces higher capillary density in newly formed tissues than the cell homing-based method [39].

The hypothesis for the current study was that decellularized human tooth scaffolds would retain their extracellular matrix integrity, support recellularization, and promote differentiation in stem cells. In our previous studies, we confirmed the effective fabrication method of decellularized human tooth scaffolds and the properties of decellularized human dental scaffolds. In addition, after recellularization of PDLSC and pulp on decellularized human dental scaffolds, their properties were confirmed in vitro [23,24]. The current study, as a follow-up study, evaluated that decellularized human tooth scaffolds promote in vivo differentiation of DPSCs and PDLSCs.

## 2. Materials and Methods

### 2.1. Tooth Sample Preparation and Cell Culture

Samples were prepared as described in a previous study [24]. Briefly, the second premolars, free of caries and restorations, were randomly collected from patients of 17 to 25 years of age under approved guidelines set by the Institutional Review Board of the Dental Hospital, Yonsei University (#IRB 2-2016-0030). Teeth were rinsed with phosphate-buffered saline (PBS) (Invitrogen, Carlsbad, CA, USA), submerged in 0.5% Chloramine T (Sigma-Aldrich, St. Louis, MO, USA) for 2 h at 4 °C, and washed in cold running water. Pulp tissue was removed with a barbed broach (Mani, Utsunomiya, Japan) to collect decellularized human periodontal ligament (dHPDL). The PDL tissue was removed with a dental currette to collect decellularized human dental pulp (dHDP). Tooth specimens were prepared using an IsoMet 1000 precision saw (Buehler Ltd., Evanston, IL, USA) as previously described [2]. A semi-cylinder-shaped saw was used for dHPDL and a doughnut-shaped saw was used for dHDP. Samples were collected in cold PBS and immediately subjected to decellularization.

Previously characterized PDLSCs and DPSCs [20,21,37,38] from three to six passages were used in recellularization experiments and were cultured in a basal cell culture alphaminimum essential medium (α-MEM) (Invitrogen) containing 10% fetal bovine serum (FBS) (Invitrogen), 1% L-glutamine/penicillin/streptomycin solution (Invitrogen), and 0.2% amphotericin B solution (Invitrogen) at 37 °C in 5% CO_2_. PDL tissues were obtained from the teeth using sterile curettes from the middle third of the teeth and pulp tissues were extirpated using a barbed broach, washed with PBS (Invitrogen), and subjected to a primary culture using the outgrowth method. PDL or pulp tissues were placed onto a 60-mm culture dish (BD Falcon, Lincoln Park, NJ, USA) and covered with a cover glass to allow the cells to grow. The cells were cultured in the culture medium described above at 37 °C in 5% CO_2_.

### 2.2. Decellularization and Recellularization

PDLSCs and DPSCs were cultured using a method from previous studies [23,24]. Tooth slices were incubated in 1% Triton X-100 (Bio Basic, Inc., Markharn, ON, Canada) for 24 h and then 1% sodium dodecyl sulfate (SDS) (Tech and Innovation, Gangwon, Korea) for 24 h to decellularize the samples. This cycle was repeated three times. These procedures were performed at room temperature with constant gentle agitation of the samples in an SH30 orbital shaker (Fine PCR, Gyeonggi, Korea) in the presence of protease inhibitor cocktail (EMD Millipore, Darmstadt, Germany). At the end of each cycle, samples were rinsed with 10% ethylenediaminetetraacetic acid (EDTA, Fisher Scientific Co., Houston, TX, USA) that had a pH level of 7.4 for 5 min and were then rinsed three times for 10 min each with PBS (Invitrogen). The tooth slices were recellularized by pipetting rat tail collagen I (Corning Inc., Corning, NY, USA) directly onto 1 × 10^7^ cells/mL of dHPDL and dHDP in 12-well culture plates (Corning Inc.). After 30 min, 1 mL of basal culture media was applied to each well and was then changed every three days. Cells were cultured at 37 °C in 5% CO_2_ for two weeks.

### 2.3. In Vivo Transplantation

In vivo procedures were performed in accordance with the protocol approved by the Institutional Animal Care and Use Committee of Yonsei University (#2016–0229). Samples were prepared using the same methods in the recellularization experiments. Decellularized dHPDL and dHDP were used as the control samples. Samples were subcutaneously transplanted into the dorsal surface of five-week-old male immunocompromised BALB/c-nu mice (SLC, Shizuoka, Japan). Four pockets were made in each mouse (*n* = 20) and either recellularized PDLSCs or recellularized DPSCs were inserted into each pocket along with decellularized dHPDL/dHDP. All transplants were retrieved after nine weeks. Of the 20 samples, six from each group were designated for histological and immunohistological analyses. The remaining 14 transplants from each group were designated for quantitative real-time polymerase chain reaction (qPCR) analysis.

### 2.4. Histology and Immunohistochemistry

Histological staining was conducted by fixing transplants with 10% formalin for 1 h, decalcifying them with EDTA, pH of 7.4 for 9 weeks at room temperature, embedding them in 4 μm-thick paraffin, and staining them with hematoxylin and eosin (HE) and Masson’s trichrome (MT) staining. IHC staining was conducted by deparaffinizing the sections in xylene, rehydrating them, and rinsing them with distilled water. Antigen retrieval was conducted by using protease K (Dako, Carpinteria, CA, USA) for cementum-derived protein 23 (CP23) (Abcam, Cambridge, UK), osteocalcin (OC) (Merck Millipore, Darmstadt, Germany) staining, and vascular endothelial growth factor (VEGF) (Abcam).Then, 10 mM of citrate buffer with a pH of 6.0 was used for cluster differentiation 34 (CD34) (Abcam) and human nuclei (HN) (Merck Millipore) staining, but this treatment was not performed for collagen type XII (ColXII) (Abcam) and dentin sialoprotein (DSP) (Santa Cruz Biotechnology, Santa Cruz, CA, USA). Sections were then immersed in 3% hydrogen peroxide for 10 min to inactivate endogenous peroxidase activity and were then incubated with primary antibodies overnight. Primary antibody information is given in Table 1. After incubation, ready-to-use EnVision + System-HRP K4001 Labeled Polymer Anti-mouse X (Dako) and ready-to-use EnVision + System-HRP K4003 Labeled Polymer Anti-rabbit (Dako) were applied for 20 min or Vectastain Elite ABC Kit (PK-6105, Vector Laboratories, Burlingame, CA, USA; goat IgG, diluted 1:200) was applied for 30 min to the samples. Color development was performed using 3,3′-diaminobenzidine substrate (Dako) and counterstained with Gill’s hematoxylin solution (Merck Millipore). Negative control sections were treated in the same manner but without primary antibodies.

### 2.5. Gene Expression Analysis by Quantitative Real Time Polymerase Chain Reaction

Gene expression levels in samples were evaluated by qPCR. Total RNA was extracted from the samples using a Qiagen RNeasy Mini Kit (Qiagen, Valencia, CA, USA) according to the manufacturer’s instructions. The integrity and concentration of extracted RNA were measured using a NanoDrop ND-2000 spectrophotometer (ThermoScientific, Waltham, MA, USA). cDNA was synthesized from 500 ng of RNA using an oligo d(T)15 primer Maxime RT PreMix kit (Intron Biotechnology, Seoul, Korea) according to the manufacturer’s instructions. A qPCR assay was performed with TB Green Premix EX Taq II (Takara Bio, Otsu, Japan) and an ABI 7300 Real-time PCR system (Applied Biosystems, Carlsbad, CA, USA) according to the manufacturer’s instructions. Primer information is given in Table 2. The expression levels of each gene were normalized to that of the gene encoding glyceraldehyde-3-phosphate dehydrogenase (GAPDH) and the relative gene expression levels were calculated using the 2^−ΔΔCt^ method [40]. Gene expression levels were calculated relative to their expression levels in the decellularized dHPDL and dHDP control samples.

### 2.6. Statistical Analyses

All experiments were performed at least in triplicate. Statistical analyses were performed with SPSS version 25.0 (SPSS, Chicago, IL, USA). The normality of the data was evaluated by using the Shapiro-Wilk test (*p* < 0.05). The Mann-Whitney U test (*p* < 0.05) was performed for all experiments using the SPSS software.

## 3. Results

### 3.1. Histological and Immunohistochemical Analysis of PDLSC Recellularization after In Vivo Transplantation

**Figure 1.**
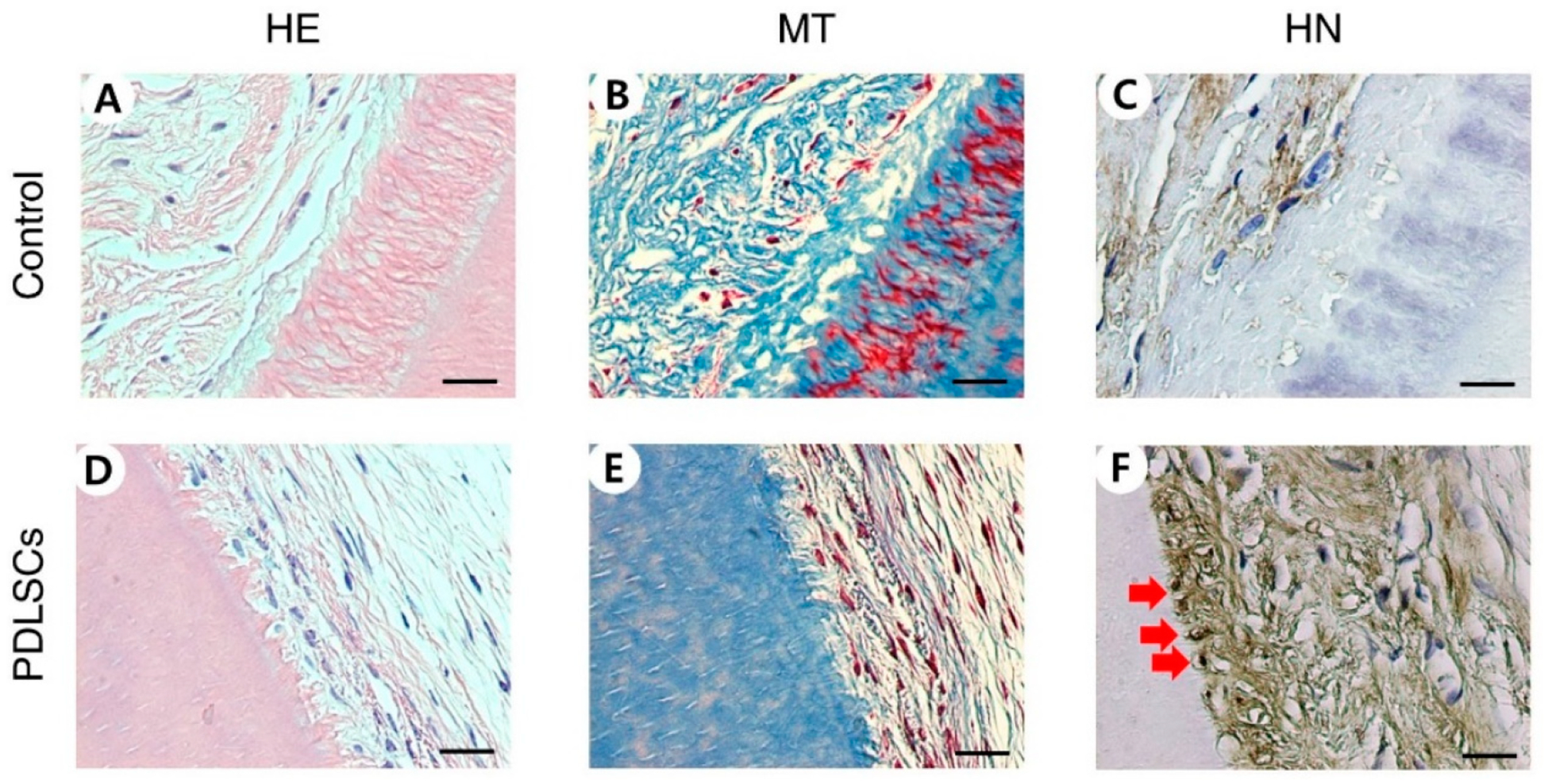
Histological characteristics of PDLSC recellularization on dHPDL transplants. HE staining (**A**,**D**), MT staining (**B**,**E**), HN staining (**C**,**F**), HE, MT, and HN staining showed that dHPDL transplants were contributed to recellularization of cells, indicated by red arrows. Scale bars: 20 μm. Abbreviations: PDLSC, periodontal ligament stem cells; dHPDL, decellularized human periodontal ligament; HE, hematoxylin and eosin; MT, Masson’s trichrome; HN, human nuclei.

**Figure 2.**
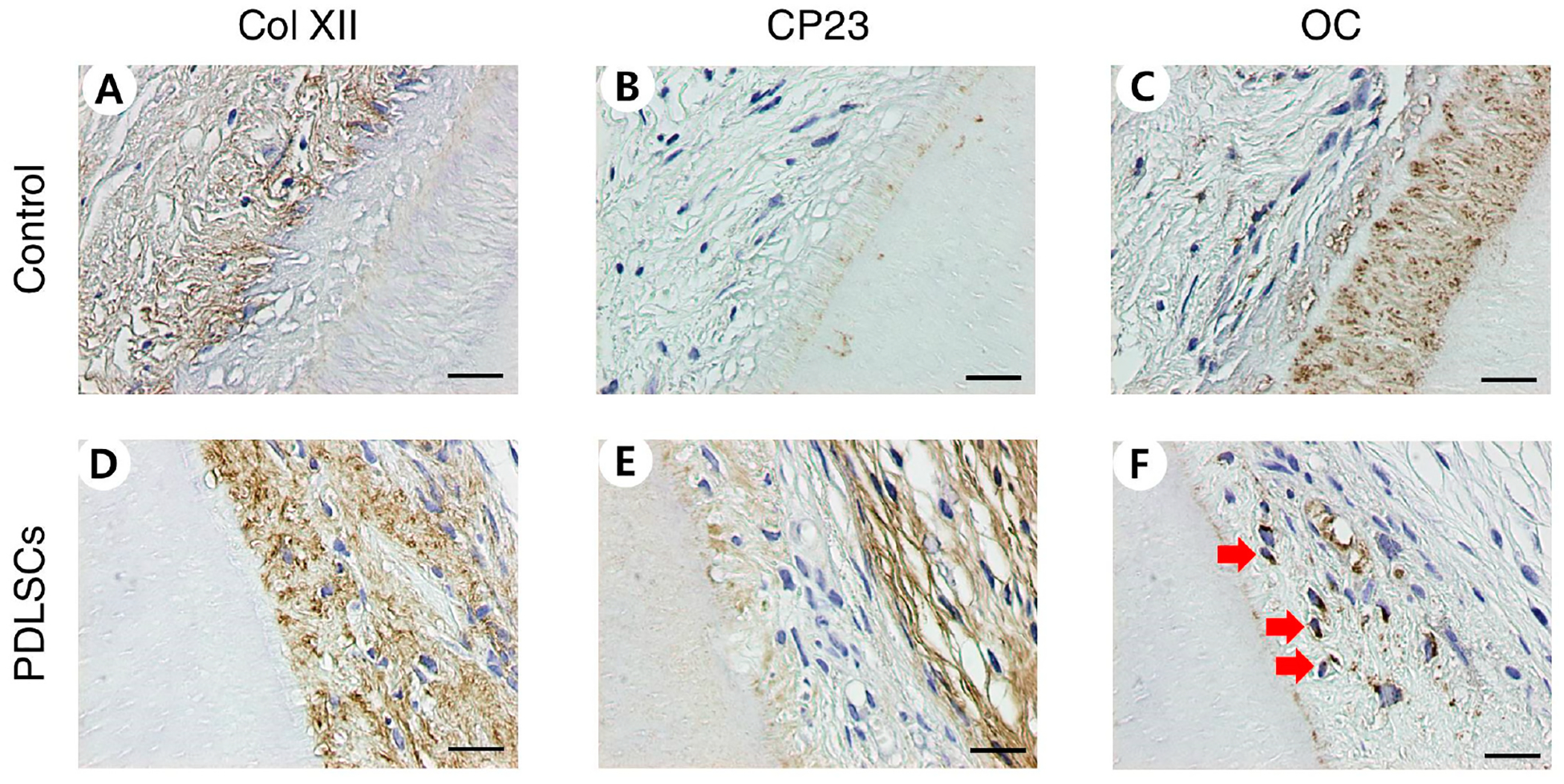
Immunohistochemical staining of PDLSCs recellularization on dHPDL transplants. dHPDL transplants were immunostained with (**A**,**D**) anti-human Col XII, (**B**,**E**) anti-human CP23, and (**C**,**F**) anti-human OC antibodies. The red arrows indicate examples of positively immunostained cells. Scale bars: 20 μm. Abbreviations: PDLSCs, periodontal ligament stem cells; dHPDL, decellularized human periodontal ligament; Col XII, Collagen type XII; CP23, cementum-derived protein 23; OC, osteocalcin.

PDLSCs were repopulated on dHPDLs and cell viability was observed after nine weeks of transplantation in mouse pockets. Cells in the control group were not inoculated, but repopulation on dHPDL ECMs was observed through HE and MT staining for the PDLSC group (Figure 1A,B,D,E). HN staining confirmed that the cells were of human origin (Figure 1C,F). Immunohistochemical staining identified anti-human Col XII, CP23, and OC expression. The PDLSC group was stained while the control group was not (Figure 2).

### 3.2. Gene Expression in Recellularized PDLSCs

**Figure 3.**
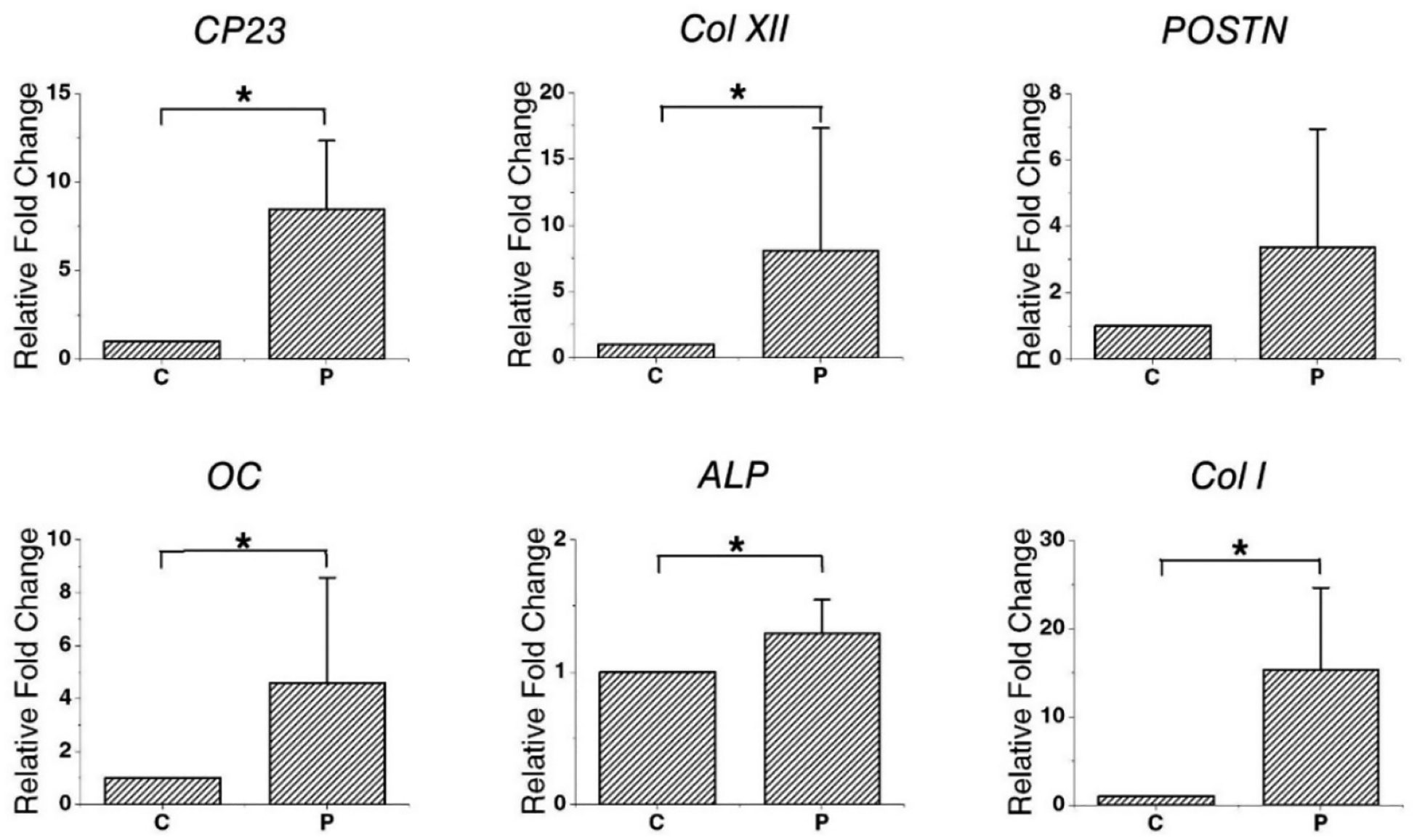
Evaluation of gene expression level by relative fold changes for genes encoding *CP23*, *Col XII*, *POSTN*, *OC*, *ALP*, and *Col I* in dHPDL transplants. The expression levels of *CP23*, *Col XII*, *OC*, *ALP*, and *Col I* differed significantly between the two groups (* *p* < 0.05). The expression of *POSTN* did not differ significantly between the two groups (*p* > 0.05). Abbreviations: C, control group; P, PDLSC group, *CP23*, cementum-derived protein 23; *Col XII*, collagen type XII; *POSTN*, periostin; *OC*, osteocalcin; *ALP*, alkaline phosphatase; *Col I*, collagen type I; dHPDL, decellularized human periodontal ligament.

qPCR was performed to assess the expression of genetic markers in recellularized PDLSCs. There was a significant difference in the expression levels of CP23, Col I, Col XII, OC, and alkaline phosphatase (ALP) between the PDLSC and control group. In particular, gene expression upregulation was observed in OC (4.59 ± 3.97-fold) and CP23 (8.46 ± 3.87-fold). However, there was no significant difference in the expression levels of periostin (POSTN) (Figure 3).

### 3.3. Histological and Immunohistochemical Analysis of DPSC Recellularization after In Vivo Transplantation

**Figure 4.**
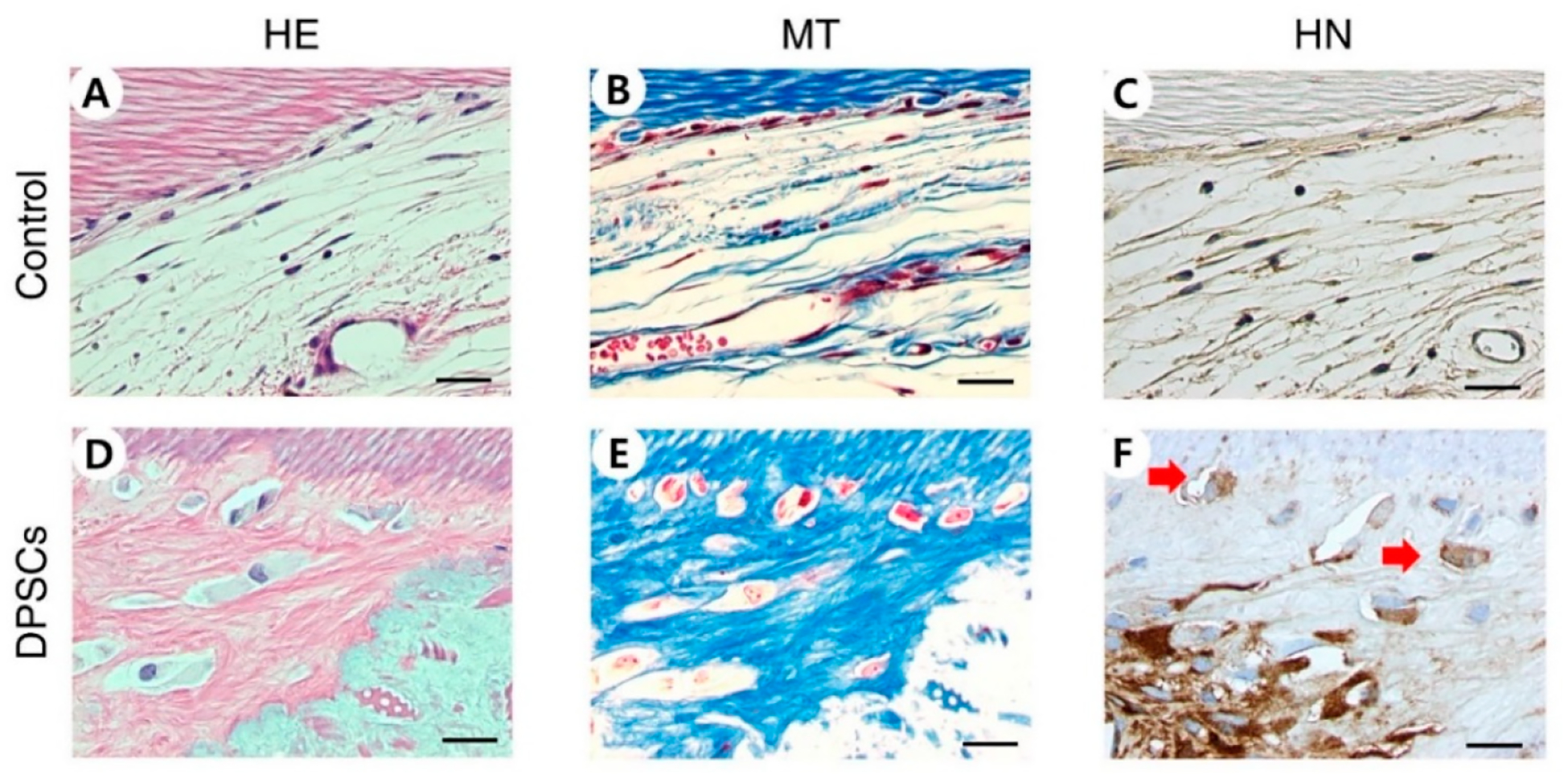
Histological characteristics of recellularized dHDP transplants with DPSCs. (**A**,**D**) HE staining. (**B**,**E**) MT staining. (**C**,**F**) HN staining. HE, MT, and HN staining showed that dHDP transplants were recellularized. Recellularized cells are indicated by red arrows. Scale bars: 20 μm. Abbreviations: dHDP, decellularized human dental pulp; DPSCs, dental pulp stem cells; HE, hematoxylin and eosin; MT, Masson’s trichrome; HN, human nuclei.

**Figure 5.**
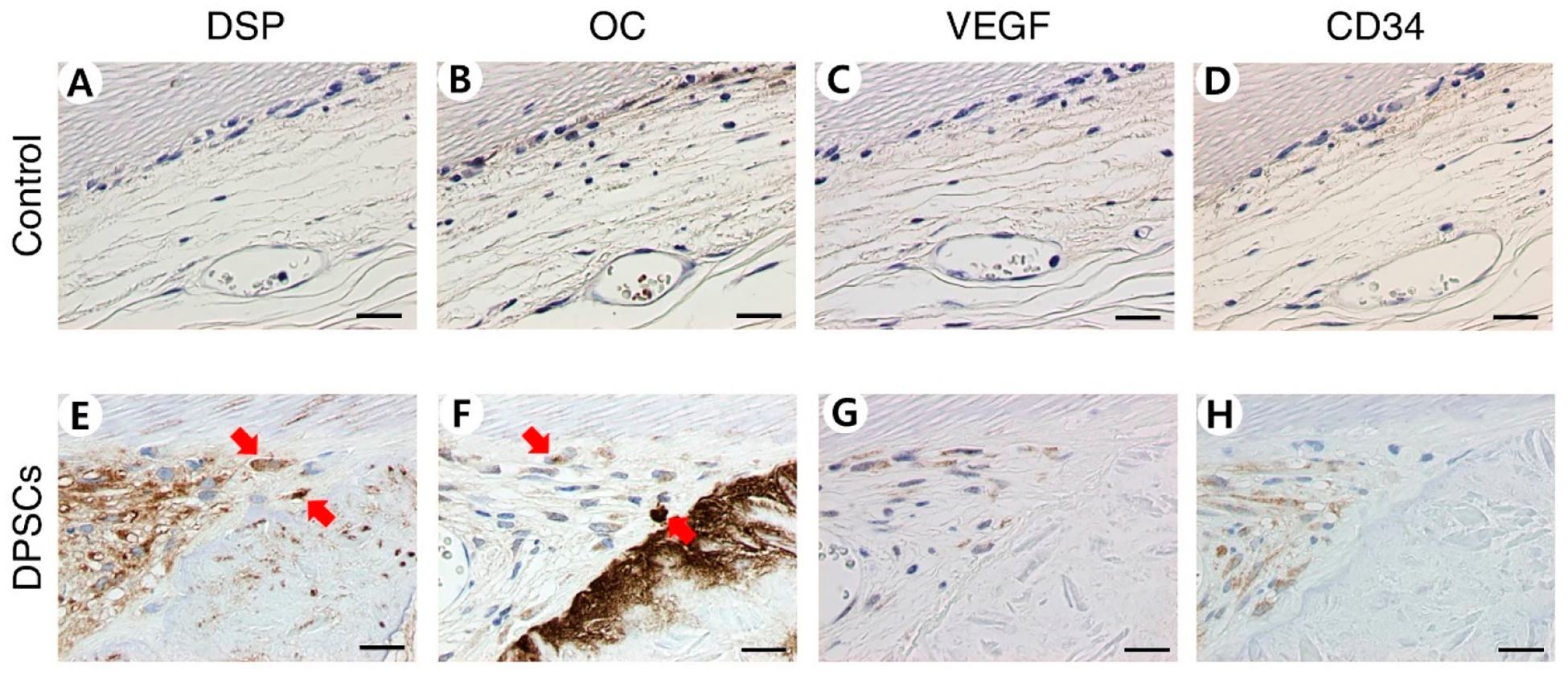
Immunohistochemical staining of dHDP transplants recellularized with DPSCs. dHDP transplants immunostained with (**A**,**E**), anti-human DSP, (**B**,**F**) anti-human OC, (**C**,**G**) anti-human VEGF, and (**D**,**H**) anti-human CD34 antibodies. Red arrows indicate positively immunostained cells. Scale bars: 20 μm. Abbreviations: dHDP, decellularized human dental pulp; DPSCs, dental pulp stem cells; DSP, dentin sialoprotein; OC, osteocalcin; VEGF, vascular endothelial growth factor; CD34, cluster of differentiation 34.

DPSCs were shown to be repopulated on dHDPs. This result was confirmed after nine weeks using the same method that was used to confirm the result for PDLs. In the DPSCs group, the repopulated cells were observed through HE and MT staining and their human origin was confirmed through HN staining (Figure 4). Unlike the control group, in the DPSC group, the newly formed hard tissues were observed through HE staining (Figure 4A,D). In the DPSC group, mineralized matrices were poorly organized and featured intermingled matrices with different alignments and embedded cells with large cytoplasmic spaces and nuclei (Figure 4D). Immunohistochemical staining showed the presence of anti-human DSP, OC, VEGF, and CD34. The DPSC group was stained while the control group was not (Figure 5).

### 3.4. Gene Expression in Recellularized DPSCs

**Figure 6.**
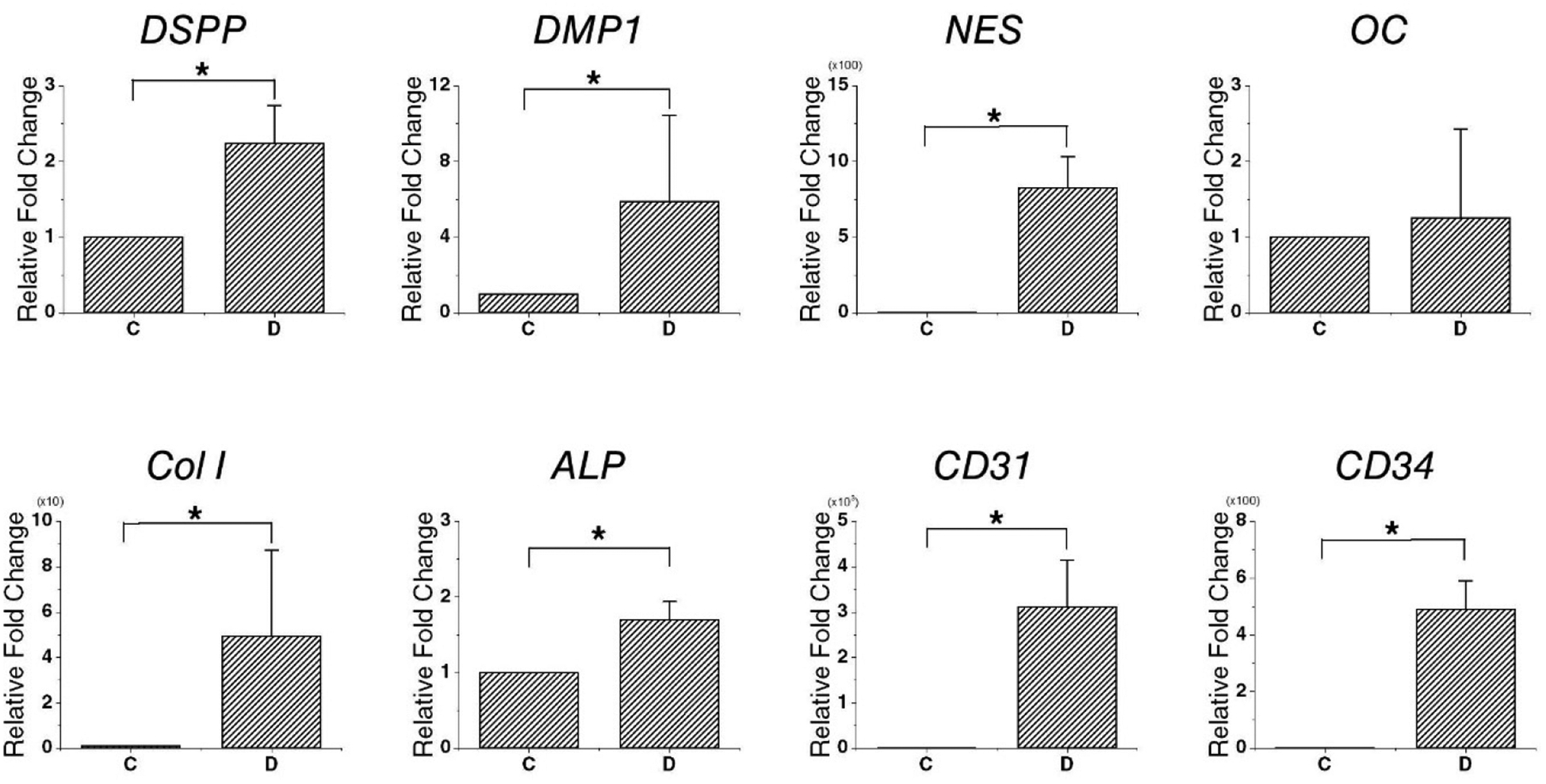
Relative expression levels of the genes encoding *DSPP*, *DMP1*, *NES*, *OC*, *Col I*, *ALP*, *CD31*, and *CD34* in dHDP transplants. Data are mean and standard deviation. The expression levels of *Col I*, *DMP1*, *NES*, *Col I*, *ALP*, *CD31*, and *CD34* differed significantly between the DPSC recellularization and control groups (*p* < 0.05). The expression of *OC* did not differ significantly between the two groups (*p* > 0.05). Abbreviations: C, control group; D, DPSC recellularization group, *DSPP*, dentin sialophosphoprotein; *DMP1*, dentin matrix acidic phosphoprotein 1; *NES*, nestin; *CD31*, cluster of differentiation 31; *CD34*, cluster of differentiation 34; dHDP, decellularized human dental pulp; DPSC, dental pulp stem cells. * idicates that there is a statistically significant difference. When *p* < 0.05, was used to show that there is a significant difference.

qPCR was conducted to confirm the expression of the relevant markers in DPSCs after recellularization. The expression of genes related to DPSCs and hard tissue, blood vessel, and nerve formation were confirmed in the DPSC recellularization group. There was a significant difference in the expression levels of DSPP, DMP1, NES, Col I, ALP, CD31, and CD34 between the DPSC recellularization group and control group. In particular, gene expression upregulation was observed in DSPP (2.24 ± 0.50-fold) and DMP1 (5.90 ± 4.54-fold) (Figure 6).

## 4. Discussion

The current study examined the cell differentiation of the stem cells inoculated in decellularized human dental tooth scaffolds. Scaffolds are an important component contributing to tissue regeneration. They provide structural support and play an important role in the cell differentiation required for new tissue to form [41,42]. Many decellularized tissues, such as skin, blood vessels, bone, and cartilage, are used as graft materials in treating patients [43,44]. Appropriately decellularized tissues preserve ECM integrity, bioactivity, spatial structures, and vascular, lymphatic, and nervous networks [45]. However, all cellular components must be removed from decellularized scaffolds as they can cause immune rejection [46]. The results of scaffold decellularization and recellularization are affected by various factors, such as the type, composition, thickness, density, and cellularity of the tissue being regenerated [47]. Previous studies have developed an optimal decellularization method and an effective method for removing residual DNA and cellular β-actin from human teeth [23,24].

The recellularization of decellularized PDL and pulp scaffolds was evaluated by qPCR and IHC staining. Stem cells repopulated on samples with pulp and PDL scaffolds but not on the control group that was not inoculated with cells. Similar to the results of in vitro experiments [24], in this study, PDLSCs were observed to be well engrafted around the cementum and they retained their unique characteristics through the high expression of the PD LSC-specific markers CP23 and POSTN. CP23 is a cementoblast marker and a regulator of the bio-mineralization of cementum [48,49]. POSTN is a PDL marker and is mainly found in cells with a mesenchymal lineage, such as osteoblasts, periodontal ligaments, and periostea [50,51]. The PDLSCs migrated deeply to the part of the semi-cylindrical dHPDLs close to the cementum by passing through the dHPDL’s collagen network (Figure 1D–F and D–F). The expression of Col XII, a marker of mature PDL, increased [52], indicating that PDLSCs differentiated into more mature PDLs. It was difficult to confirm the newly formed hard tissue through HE staining, but OC and ALP, which are involved in the mineralization of cementum and bone, were expressed [53,54]. During recellularization, PDLSCs moved close to the cementum of dHPDL, which affected the generation of mature PDL, and caused the differentiation of hard tissues in vivo. These results indicate that transplanting stem cells onto scaffolds can help repair or regenerate damaged periodontal tissue.

Recellularization on DPSCs had different characteristics than recellularization on PD LSCs. In the DPSC recellularization group, the formation of newly formed hard tissue was shown through HE staining. The newly created hard tissue did not have a dentin-like structure, but the hard tissue-related markers DSPP, DMP1, ALP, OC, and Col I were expressed [54–58]. In addition, nestin, which is related to nerves, and CD31 and CD34, which are related to angiogenesis, were expressed, indicating that hard tissue, nerves, and blood vessels may have been generated. Vascularization is an important part of tissue regeneration because blood vessels supply essential elements for the process. Angiogenesis was observed to have occurred through the expression of VEGF shown by IHC and the expression of CD31 and CD34 shown by qPCR. VEGF is a marker for angiogenesis, which accompanies osteogenesis. The generation of blood vessels facilitates the supply of nutrients, oxygen, and minerals essential for mineralization and bone-forming progenitors. Osteogenic factors, such as BMP2, promote osteoblast differentiation and mineralization and are secreted from blood vessels [59,60]. Mature osteoblasts generate mature angiogenic factors, such as VEGF, which also promote angiogenesis. Thus, the presence of these factors indicate that processes are occurring which promote tissue regeneration. Furthermore, when endothelial cells are co-seeded with stem cells on scaffolds, the roles of factors that can positively affect osteogenesis and vascularization can be expected [61].

Teeth are complex organs made up of various cells and structures, thus it is difficult to repair once damaged PDL and pulp, which play an important role in teeth’s physical, formative, nutritional, and sensory functions and affect tooth vitality [62,63]. PDL is a soft connective tissue that connects the root of the tooth and the inner wall of the alveolar socket [62]. PDL can be damaged by local factors, such as plaque, dental calculus, and traumatic occlusion, and destroyed by systemic or external factors, such as smoking. PDL can also be damaged by external force, more so by avulsion than luxation. Damage to PDL can cause early tooth loss or root resorption, thus recovery of damaged PDL is essential for tooth recovery [64,65]. Pulp can become necrotized due to caries, trauma, and anatomic variations, such as dens evaginatus and dens invaginatus [66]. The traditional treatment for necrotized pulp is to remove and replace it with dental material, which can harm the tooth vitality. The loss of vitality can disrupt the development of immature teeth, resulting in a poor clinical prognosis. REP development has made a significant progress in recent years, becoming an alternative treatment for damaged pulp that can preserve tooth vitality [36]. Although REP promotes continuous tooth growth and development of immature teeth, and it guides endodontic repair through cell-homing rather than the true regeneration of the pulp tissue [67].

Contemporary dental treatment processes simply replace missing tooth structures or entire missing teeth through various methods, including direct restoration and the insertion of inert dental materials, fixed prostheses, and dental implants. The development of osseointegrated dental implants was a revolutionary advance in dental treatment, making it possible to recover a missing tooth without affecting surrounding healthy teeth [68]. However, even biologically advanced materials and mechanically advanced implants still suffer structural limitations and cannot mimic biological functions. Dental tissue therapies are transitioning from traditional methods to working with patient biology, which is increasing treatment success rates [69]. The goal of traditional dental treatments is to restore the function and morphological integrity of the damaged tooth. However, the goal of cutting-edge dental treatments is to maintain tooth vitality and regenerate tissue. To overcome these limitations, research is being conducted to develop regenerative clinical treatments [70]. The ultimate goal of this line of research is to regenerate entire teeth. A study on the manipulation of a bioengineered incisor tooth germ using single cells isolated from the epithelium and mesenchyme of the dental germ produced the successful eruption of a bioengineered tooth germ. Bioengineered teeth exhibit masticatory properties and respond to harmful stimuli [71,72].

Previous studies on the use of stem cells in tooth regeneration have only used specific epithelial and mesenchymal stem cells. Although some of the mechanisms behind tooth development have not yet been identified, scaffolds can play an important role in tooth regeneration in addition to stem cells and growth factors [73]. The decellularized human tooth scaffolds used in this study, maintained structure with remaining collagen from both pulp and PDL scaffolds. Both types of scaffolds were recellularized when cultured in vivo after being inoculated with DPSCs and PDLSCs. These stem cells expressed genes related to pulp and PDL regeneration. Mesenchymal stem cells applied to decellularized human tooth scaffolds have the potential to open a novel approach to pulp and PDL regeneration through proliferation and differentiation. Therefore, a single scaffold could contribute to regenerate both PDL and pulp by recellularization of PDLSCs and DPSCs.

This study has limitations in that it may show structural limitations due to the different area of PDL and pulp chamber size for each scaffold. The results exceeded the structural and functional limitations seen in scaffolds such as tissue substitutes, bioactive substances, and synthetic scaffolds used in the existing dental tissue regeneration studies. However, the process of restoring vitality of teeth by using damaged natural teeth as a scaffold still has many factors that can affect the results, such as the stem cell inoculation method and the degree of tooth damage, and further research is needed.

## 5. Conclusions

The current study showed that decellularized human teeth can be used as scaffolds to regenerate PDL and pulp using PDLSCs and DPSCs. PDLSCs and DPSCs inoculated into decellularized human tooth scaffolds and cultured in an in vivo environment differentiated into their corresponding tissues and express specific relevant genes.

## Figures and Tables

**Table 1.** Primary antibodies for immunohistochemistry.

Antibodies	Catalog Number	Host Species	Dilution Factor
CD34	Ab110643	Rabbit	1:100
Col XII	Sc-68862	Rabbit	1:2000
CP23	Sc-164031	Goat	1:500
DSP	Sc-33586	Rabbit	1:500
HN	MAB1281	Mouse	1:100
OC	AB10911	Rabbit	1:8000
VEGF	Ab183100	Goat	1:100

Abbreviations: CD34, cluster of differentiation 34; Col XII, collagen type XII; CP23, cementum-derived protein 23; DSP, dentin sialoprotein; HN, human nuclei; OC, osteocalcin; VEGF, vascular endothelial growth factor.

**Table 2.** qPCR forward and reverse primer sequences. The annealing procedures were performed at 60 °C for all primers.

Gene	Forward Primer Sequence (5′–3′)	Reverse Primer Sequence (5′–3′)
*ALP*	GGACCATTCCCACGTCTTCAC	CCTTGTAGCCAGGCCCATTG
*CD31*	CCCATTGTTCCCGGTTTCCA	AGTTAGTTCTGCCTTCGGGC
*CD34*	CGCTGCCTTGCCAAGACTAA	CCTAGAGAGACGCACCGAGT
*Col I*	CGATGGCTGCACGAGTCACAC	CAGGTTGGGATGGAGGGAGTTTAC
*Col XII*	CGGACAGAGCCTTACGTGCC	CTGCCCGGGTCCGTGG
*CP23*	AACACATCGGCTGAGAACCTCAC	GGATACCCACCTCTGCCTTGAC
*DMP1*	GATCAGCATCCTGCTCATGTT	AGCCAAATGACCCTTCCATTC
*DSPP*	GGGATGTTGGCGATGCA	CCAGCTACTTGAGGTCCATCTTC
*NES*	GCCCTGACCACTCCAGTTTA	GGAGTCCTGGATTTCCTTCC
*OC*	CAAAGGTGCAGCCTTTGTGTC	TCACAGTCCGGATTGAGCTCA
*POSTN*	CACAACCTGGAGACTGGAC	TGTCTGCTGGATAGAGGAG
*GAPDH*	TCCTGCACCACCAACTGCTT	TGGCAGTGATGGCATGGAC

Abbreviations: *ALP*, gene encoding alkaline phosphatase; *CD31*, gene encoding cluster of differentiation 31; *CD34*, gene encoding cluster of differentiation 34; *Col I*, gene encoding collagen type I; *Col XII*, gene encoding collagen type XII; *CP23*, gene encoding cementum-derived protein 23; *DMP1*, gene encoding dentin matrix acidic phosphoprotein 1; *DSPP*, gene encoding dentin sialophosphoprotein; *NES*, gene encoding nestin; *OC*, gene encoding osteocalcin; *POSTN*, gene encoding periostin; *GAPDH*, gene encoding glyceraldehyde-3-phosphate dehydrogenase.

## Data Availability

Data are contained within the article.
